# A method for the production of large volumes of WAF and CEWAF for dosing mesocosms to understand marine oil snow formation

**DOI:** 10.1016/j.heliyon.2017.e00419

**Published:** 2017-10-10

**Authors:** Terry L. Wade, Maya Morales-McDevitt, Gopal Bera, Dawai Shi, Stephen Sweet, Binbin Wang, Gerado Gold-Bouchot, Antonietta Quigg, Anthony H. Knap

**Affiliations:** a Geochemical and Environmental Research Group, Texas A&M University, 833 Graham Road, College Station, Texas 77845, USA; b Department of Marine Biology, Texas A&M University at Galveston, PO Box 1675, Galveston, TX 77553, USA

**Keywords:** Microbiology, Biological sciences, Environmental science, Earth sciences, Natural sciences

## Abstract

Marine oil snow (MOS) formation is a mechanism to transport oil from the ocean surface to sediments. We describe here the use of 110L mesocosms designed to mimic oceanic parameters during an oil spill including the use of chemical dispersants in order to understand the processes controlling MOS formation. These experiments were not designed to be toxicity tests but rather to illustrate mechanisms. This paper focuses on the development of protocols needed to conduct experiments under environmentally relevant conditions to examine marine snow and MOS. The experiments required the production of over 500 liters of water accommodated fraction (WAF), chemically enhanced water accommodated fraction of oil (CEWAF) as well as diluted CEWAF (DCEWAF). A redesigned baffled (170 L) recirculating tank (BRT) system was used. Two mesocosm experiments (M1 and M2) were run for several days each. In both M1 and M2, marine snow and MOS was formed in controls and all treatments respectively. Estimated oil equivalent (EOE) concentrations of CEWAF were in the high range of concentrations reported during spills and field tests, while WAF and DCEWAF concentrations were within the range of concentrations reported during oil spills. EOE decreased rapidly within days in agreement with historic data and experiments.

## 1 Introduction

On April 20, 2010, in the northern Gulf of Mexico, the deep-sea petroleum-drilling rig Deepwater Horizon (DWH) exploded and oil was released over the next 87 days, releasing approximately 4.1 million barrels of Sweet Louisiana Crude Oil (later judged to be 3.19 million barrels) and 205,000 Mt of methane into the water column at a depth of 1500 m (Graham et al., 2010; Harlow and Brantley Harlow, 2011; Bælum et al., 2012). Both were ejected at a considerable rate, leading to the formation of small oil-droplets (Joye, 2015). A deep-water oil plume from 900 to 1200 m below the sea surface formed as a consequence of the depth and elevated pressure of the blowout and the interaction between oil and gas and the solubility of each component (Camilli et al., 2010; Diercks et al., 2010; Joye, 2015). A considerable amount of oil also reached the surface. The spatial and temporal distribution of water column hydrocarbon concentrations measured during and after the spill have been well documented (Wade et al., 2016).

The spill response included physical removal of the oil by pumping, skimming, and burning. Initially the dispersant COREXIT 9527 was used, however most of the 37,500 barrels of dispersant was COREXIT 9500A sprayed on the surface of the ocean as well as injected directly into the oil plume at the wellhead (Bælum et al., 2012). The effects of dispersant use on the oil, and specifically on the polycyclic aromatic hydrocarbons (PAH), a toxic fraction of oil on the marine environment (Diercks et al., 2010; Bælum et al., 2012) is a concern. Shortly after the DWH oil spill, Passow et al. (2012) observed profuse flocs of mucus-abundant marine oil snow with oil droplet inclusions were observed floating on the surface and termed marine oil snow (MOS). The mucus associated within the marine snow was measured as transparent exopolymer particles (TEP) produced by microbes (Passow et al., 2012). Less than a month later, the marine snow had disappeared from the surface water (Passow et al., 2012; Ziervogel et al., 2012) leading to the hypothesis that the MOS was formed *in situ* in the presence of oil, and eventually sunk into deeper waters (Passow et al., 2012). A better understanding of this important phenomenon’s contribution to the removal and degradation of oil during spills is needed. One approach is to determine the mechanism of marine snow formation by duplicating the process through experimental mesocosm studies. This paper focuses on the development of protocols needed to conduct experiments under environmentally relevant conditions to examine marine snow and MOS. Future papers will describe marine snow formation mechanisms and biodegradation.

The objective of the larger overall study was to develop a mechanistic understanding for the interactions of oil with and without dispersant with exopolymeric substances (EPS) under various environmental conditions (Quigg et al., 2016). The hypothesis is that bacteria and phytoplankton respond to oil and oil plus Corexit by producing EPS, TEP and/or marine snow, which interact with minerals, organic particles and organisms consequently influencing the fate, distribution and potential effects of hydrocarbon contaminants. In addition, it proposes that in the presence of oil and/or Corexit, some members of the microbial community will break down hydrocarbons as a means of obtaining their source of carbon and energy. Generally, oil toxicity studies are criticized as employing unusually high concentrations of oil which is needed to illicit a biological response, or the tests are carried out in closed containers and do not represent the ocean environment (Aurand and Coelho, 2005; Lee et al., 2013). It is also clear that field experiments provide the most realistic information (Ballou et al., 1989); however it is very difficult to receive the permits requested to spill oil in the environment. Mesocosms are therefore considered as valuable tools for ecological research as well as good surrogates for environmental risk assessments (Odum, 1964; Ives et al., 1996; Nordtug et al., 2011). The scales vary from “beakers to bay” (Strickland, 1967).

**Fig. 1:**
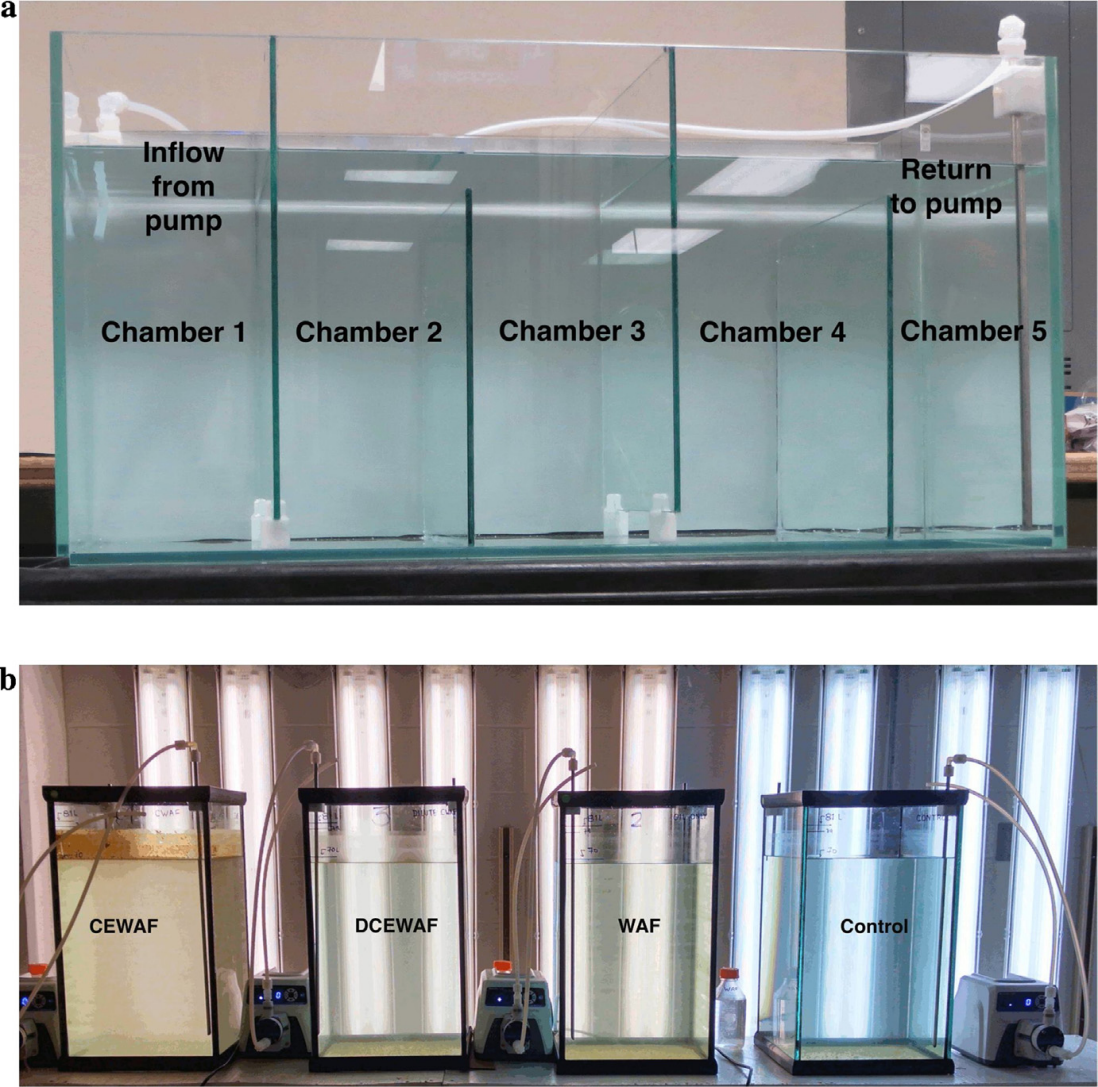
(a) Baffled Recirculating Tank. Each chamber is numbered chambers 1–5 from the left; (b) 110L Mescosm tanks used for Marine Oil Snow experiments.

**Fig. 2:**
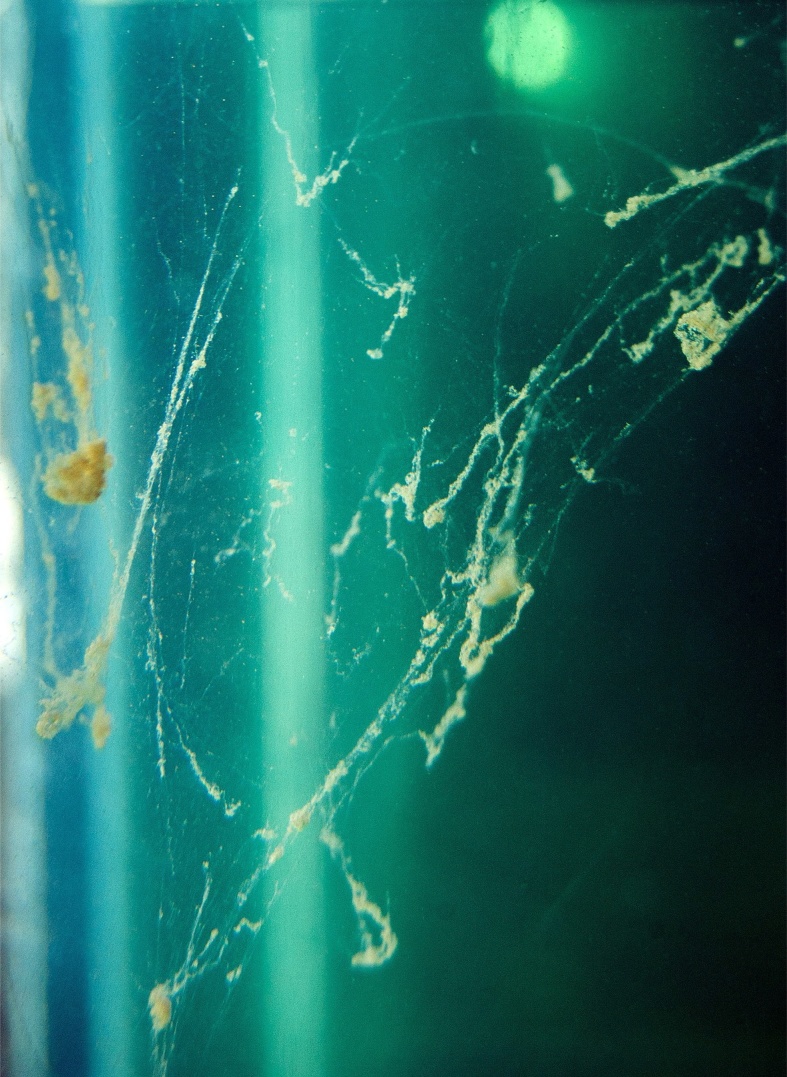
MOS forming in WAF tank in Mesocosm experiment 2.

One research objective was to study the mechanism of EPS/TEP formation in the presence of oil and oil plus dispersant using mesocosms. Another objective was to have dosing concentrations of WAF and CEWAF reflecting what might reasonably be expected to be produced and occur in the marine environment during a spill event. There are tradeoffs between working at smaller scales with greater control versus larger scale experiments, which better reflect the actual environment. Here we describe the design and use of baffled recirculating tanks (BRT) for the production of large volumes of WAF, DCEWAF and CEWAF for dosing of medium scale mesocosms (110 L). BRTs (Fig. 1a) were adapted from a system described by Knap et al. (1983). In the present study the BRT systems were necessary to produce large quantities of WAF and CEWAF. The CROSERF method (Aurand and Coelho, 2005), would be impractical for the large volumes needed for our mesocosm tanks (Fig. 1b). Nordtug et al. (2011) have produced an elegant system for dosing in ecotoxicology studies, however it produces dispersed oil for 14 (5 L) dosing vessels (total 70 L). The tanks we needed to use for MOS studies were 96 L in the first mesocosm study (M1) and modified to 110 L in the second experiment (M2). Therefore we needed to produce over 500 L of WAF and CEWAF at the same time so experiments could be run in triplicate and provide sufficient EPS for detailed chemical and biological studies on marine snow and MOS (Fig. 2).

## 2 Materials and methods

### 2.1 Baffled recirculating tanks (BRT)

The WAF and CEWAF for these experiments were produced using Macondo Surrogate oil (MSO) (specific gravity 0.86 g/ml) provided by BP. The Macondo Surrogate oil was from the Marlin Platform Dorado (SO-20120211-MPDF-003) and is similar to the DWH Oil. The CEWAF was prepared by premixing Corexit EC9500A (specific gravity 0.949 g/ml) with Macondo Surrogate oil at a ratio of 1:20 (V/V), which is in the lower end of the field application recommended range of 1:10 to 1:50 (USEPA, 1995).

The BRT constructed of fiberglass by Knap et al. (1983) was useful at the time for providing a flow-through system for dosing corals with high concentrations of oil and chemically dispersed oil for 24 h. There was no concern of interference from plasticizers etc. due to the high concentrations of oil (20 mg/l) and the analyses of EPS was not undertaken. In the case of the present study target concentrations of WAF ranged from 0.2 to 2 mg/l and the mechanisms of MOS formation requires very sophisticated microbiology and chemistry (Quigg et al., 2016). The mesocosms and BRT were made solely of glass due to concerns of interference with EPS chemical measurements. The BRT glass tanks were 43 × 88 × 44 cm with 4 glass baffles. The baffles were held in place with a minimal amount of silicone adhesive on the sides and supported by machined stainless steel supports. We found no interference from the silicone in our analysis (data not shown). The baffles were placed in each of the tanks so that water passed under the first and third baffles and over the second and fourth baffles (Fig. 1a). This allows water non-accommodated oil to rise to the surface of the tanks. The BRT total capacity is 170 L, and allowing water to be added to a level of 1 cm above each baffle results in a total water volume of 130 L. Our experiments required the construction of 6 baffled tanks allowing for the production of over 500 L of WAF and CEWAF to be made within 24 h. A Masterflex^®^ PTFE-Diaphragm Pump with Teflon heads and tubing was used to recirculate the water in each of the BRT at 350 ml per min. Water was drawn from the bottom of the last chamber and pumped back to 5 cm below the water surface of the first chamber (Fig. 1a). One magnetic stirrer was placed below the tanks to provide energy in sections 3 and 4 at 60 rpm. The water was fully recycled in approximately 6 h. An Arrow 1750 stirrer with a stainless steel rod and propeller was placed in the first chamber to provide mixing energy 20 cm below the water surface. The stirrer was set to a speed such that there was only a shallow vortex when oil was being added. For M1 (the pilot study) the BRT was stirred at a higher setting with a deeper vortex (setting 4 on the Arrow 1750) to mix every two to three hours so that the oil would be mixed deeper into the water. M2 was stirred at a slower speed (Setting 2 on the Arrow 1750) to provide lower concentrations of oil in water.

### 2.2 Droplets size distribution measurements

**Fig. 3:**
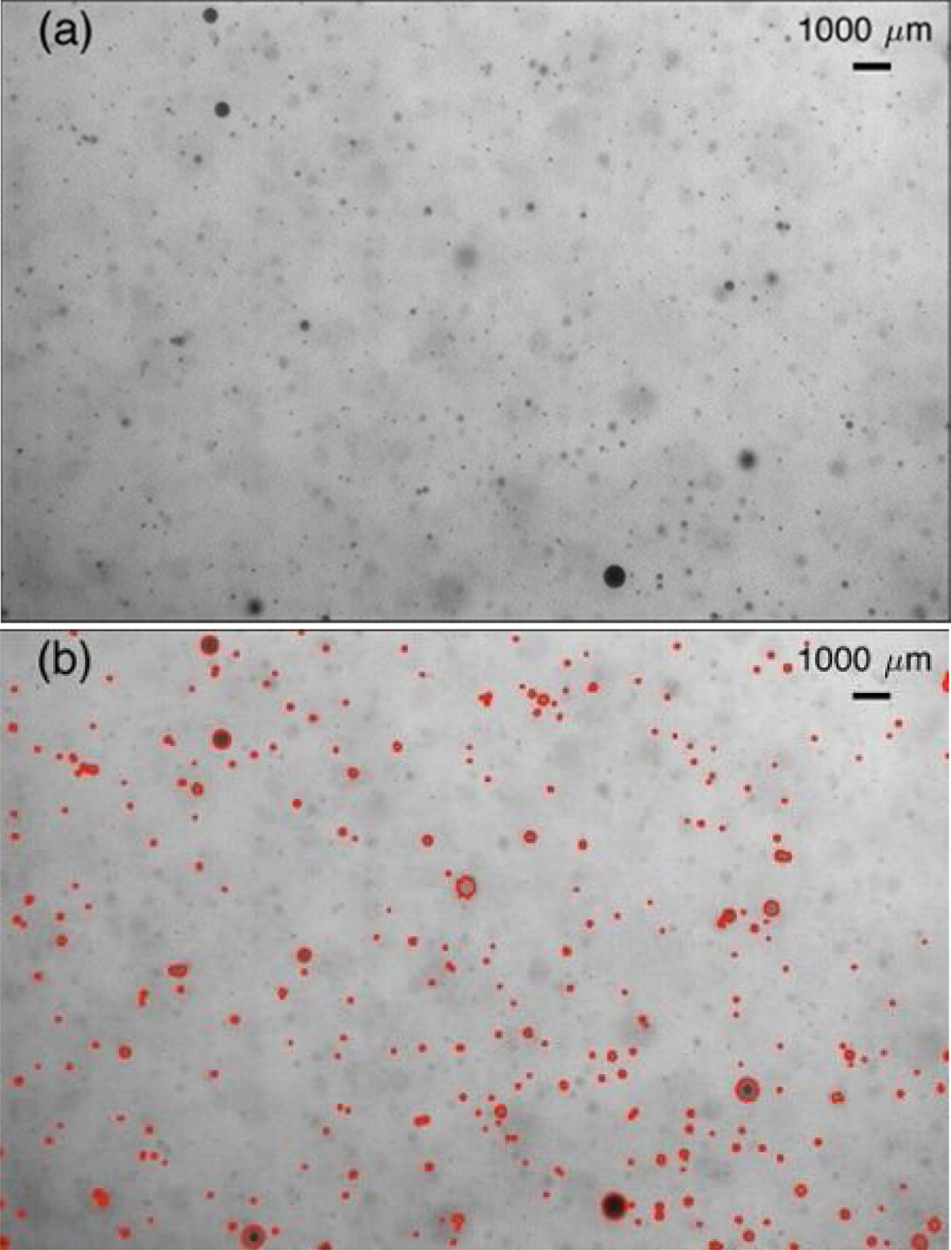
(a) A raw sample image of oil droplets; (b) The result of identification and sizing of in-focus oil droplets.

The size distributions of oil droplets were measured during WAF and CEWAF production using a high-speed camera (Phantom Miro M340, Vision Research) with a 200 mm micro-lens (Nikon Nikkor). Backlighting was used to generate dark oil droplet images in a white uniform background (see Fig. 3a), permitting automated particle identification and size measurements (Wang and Socolofsky, 2015a, 2015b).

For only the droplet size experiments, Instant Ocean seawater (30 PSU) was used with Macondo Surrogate oil (25 ml) added to the BRT using the same protocols described above. Three sets of image data were obtained during the experiment, where the droplet images were taken at the mid-depth of the BRT. The first set of measurements took place immediately after the oil release, which is labeled as *t*_0_. The oil droplet images were taken in chambers 2, 3, 4, and 5 of the BRT (see Fig. 1a) sequentially, within 12 min after the oil was released. There were repeated imaging measurements for each chamber of the BRT at 1 h (*t*_1_) and 3 h (*t*_2_) after the oil was released.

For each measurement, 2020 images were obtained at 24 frame-per-second (fps), which spanned about 84 seconds of image data. The image calibration showed the resolution of image was about 9.9 μm/pixel, providing a full image field of view (FOV) of 25.3 × 15.8 mm^2^. The exposure time was set to 500 μs to eliminate motion blur.

### 2.3 Mesocosm study 1

The seawater used in the Mesocosm 1 experiment (M1) was collected on July 30, 2015, 8 kilometers off-shore south of Galveston (TX) in the Gulf of Mexico. The seawater (34 PSU) was processed through a charcoal filter to remove large particulates and debris. Four mesocosm tanks were treated in the following way. The control tank was filled with the seawater directly from the storage tank. This seawater was also used to fill two 130 L Baffled Recirculating Tanks (BRT’s) for WAF and CEWAF production. The WAF was prepared by mixing a total of 24 ml (2 ml to start, 2 mL after 1 h, then 5 ml at ∼ 2, 3, 4 and 5 h) of Macondo Surrogate oil (MSO) into 130 L of the seawater. Total mixing time from the start of oil addition to transfer to the mesocosms was 14 h. The WAF (79 L) was transferred to a tank and mixed. CEWAF production involved mixing Corexit 9500A with MSO in a ratio of 1:20 (Corexit to oil, V/V) and 24 ml of this mixture (2 ml to start, 2 ml after 1 h, then 5 ml at ∼ 2, 3, 4 and 5 h total of 24 ml) was added to 130 L of seawater and mixed for 24 h. DCEWAF mesocosm treatment was produced by adding 9 L of CEWAF to 70 L of the original seawater for a total volume of 79 L.

Plankton (≥63 μm) samples were collected just prior to use at from the dock at Texas A&M Galveston Campus using a net and transferred into polycarbonate bottles. This concentrated plankton sample was introduced to each mesocosm and stirred (2 L to each tank for a final volume of 81 L) immediately prior to starting the experiments. Banks of lights were placed behind each of the glass mesocosm tanks and a 12:12 light/dark cycle employed. For M1 sampling for microbial, biological and chemical analysis was through a 0.4 cm diameter stainless steel tubing placed ∼5 cm from the bottom of each tank. A Masterflex^®^ PTFE-Diaphragm Pump with Teflon heads and tubing were used for sampling. Samples were collected at periodic time points over a 112 h period.

### 2.4 Mesocosm 2

Mesocosm 2 was designed with a system similar to M1 but this time there were 3 replicate tanks per treatement with slightly larger tanks (110 L) with Teflon sample valves fitted with external silicon O-rings 10 cm from the bottom of each tank. A PTFE stopcock attached to Teflon tubing was used for sampling. It was determined that this gravity driven sampling would be preferable to the pumped sampling system used in M1. Seawater (31 PSU) was collected from the same location and treated as that used in M1, on October 17, 2015. Control tanks were filled with seawater from the storage tank. WAF was prepared by mixing 25 ml (5 ml ∼ every 30 min for 2.5 h) of MSO into 130 L of seawater then mixing for 12 to 24 h. The WAF was then introduced into the WAF mesocosm tanks and filled to 87 L with the original seawater. For the CEWAF, Corexit was mixed with oil at a ratio of 1:20 and 25 ml of this mixture (5 ml every 30 min for 2.5 h) of MSO plus Corexit were added to 130 L of seawater, which was mixed for 24 h prior to being transferred to the mesocosm tanks. DCEWAF was prepared by mixing 9 L of CEWAF with 78 L of the original seawater for a total volume of 87 L. Plankton (≥63 μm) were collected from the TAMUG dock using a net and transferred into polycarbonate bottles. This concentrated plankton sample was introduced to the tanks and stirred (2 L to each mesocosm, for a final volume of 89 L) immediately prior to starting the experiments. Banks of lights were placed behind each one of the glass tanks and a 12:12 light dark cycle used. Samples were collected at the start of the experiment and every 24 h up to 72 h.

### 2.5 Estimated oil equivalents (EOE)

The estimated oil equivalents (EOE) were determined by fluorescence (Wade et al., 2011) using Macondo Surrogate oil as the calibration. Water samples (5 to 20 ml) were extracted with 5 ml of dichloromethane. An aliquot of the extract was placed in a cuvette for fluorescence analyses with a Horiba Scientific Aqualog Fluorometer (excitation 254 nm; emission 365 nm). The EOE were determined from the calibration curve (Wade et al., 2011). Samples with florescence responses exceeding the highest calibration standard were diluted until the response was within the calibration range. Samples were collected periodically throughout the course of the experiment.

### 2.6 Analysis of PAHs

Water samples (1 to 3.5 L) were extracted with dichloromethane in a separatory funnel, spiked with appropriate amounts of deuterated compounds as surrogate standards (e.g., d8- naphthalene, d10-acenaphthene, d10-phenanthrene, d12-chrysene, and d12-perylene). GC internal standards (e.g., d10-Fluorene and d12- Benzo(a)pyrene) were added to the DCM extracts and the final volume was reduced to 1 ml. MSO samples were diluted and analyzed after addition of surrogate standards.

The analyses was on an Agilent 6890 gas chromatograph coupled with an Agilent 5973 mass selective detector (GC-MS). Separation of PAHs was accomplished with a DB-5 MS fused silica capillary column (30 m × 0.25 mm i.d., 0.25 μm film thickness, Agilent). The oven temperature was programmed to increase from an initial temperature of 60 °C to 150 °C at 15 °C/min, then at 5 °C/min to 220 °C, and finally at 10 °C/min to a final temperature of 300 °C with a final holding time of 10 min. PAHs were identified based on the comparison of the retention time and ratios of selected ions with those in the calibration standards. Quantitation was accomplished by adding surrogate compounds to water oil extracts. Results were reported in ug/l for water samples and mg/g for oil samples. The percent distributions were calculated by dividing the specific PAH concentration by the total PAH concentration.

## 3 Results and discussion

### 3.1 Droplet size in the BRT

The droplet size of oil in WAF and CEWAF treatments is important as this controls the distribution of oil in the water column. The evolution of droplet size with time in the chambers of the BRT following oil addition under the normal experimental conditions was determined. We did not measure the light intensity throughout the experiment. However, during the course of imaging, the light intensity can be estimated from the images. The intensity value of every pixel in each image was averaged to provide an overall assessment of the light intensity transported through the water. Due to the abundance of oil droplets in the water, the intensity reduction was observed from images before and after oil being added. The intensity reduction was also visually observed. In order to minimize the impact of non-uniform background intensity, an adaptive thresholding method using MATLAB Imaging Processing Toolbox (Mathworks Inc., 2014) converting the intensity images to binary images for droplet size analysis was applied. An appropriate threshold was selected to distinguish the in-focus oil droplets from the background and out-of-focus oil droplets. The minimum measurable droplet size was approximately 35 μm. The raw images (Fig. 3a) were processed and the result after image processing (Fig. 3b) were used to determine droplet size distribution. The out-of-focus oil droplets (i.e., larger and lighter dark dots Fig. 3a) were excluded in the droplet identification process. Because most of the oil droplets are spherical due to their small size (Clift et al., 1978), the equivalent spherical droplet diameters were calculated.

Immediately after oil release, we observed that a large amount of oil droplets were transported from chamber 1 to chamber 2. The overall droplet sizes decreased from chamber 2 to chamber 5 as the result of weakened transport of larger droplets due to weaker mixing along the downstream direction. With increasing time the number of droplets decrease in the tank due to surfacing of buoyant oil droplets, re-forming an oil slick at the air-water interface. Oil slicks were observed in all of the downstream chambers demonstrating the immiscible nature of the oil (oil was in excess to its solubility).

**Fig. 4:**
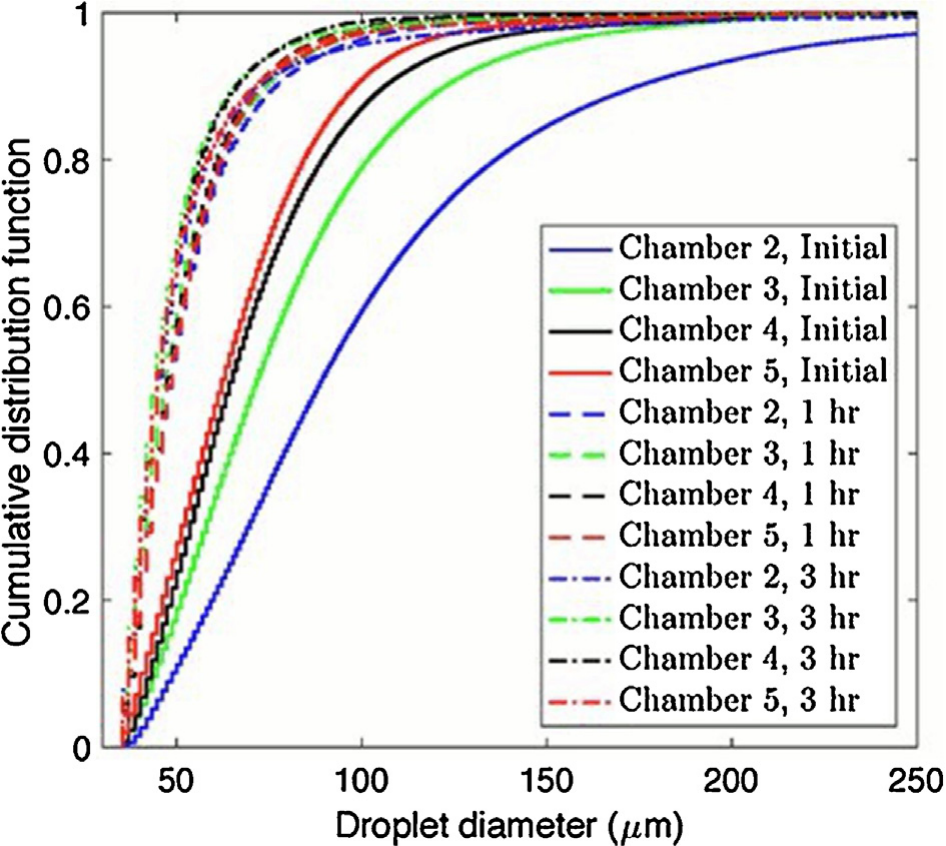
Cumulative distribution function of droplet sizes in different parts of the tank at different times after addition of oil.

After oil release, we observed a clear shift of oil droplets toward smaller size from chamber 2 to chamber 5. One hour after oil addition, the cumulative droplet size distributions was similar in all chambers (see Fig. 4). The differences of cumulative distribution of droplet sizes among different chambers of the tank were similar three hours after the oil addition. The cumulative droplet size distribution did not change significantly from one hour to three hours after oil release suggesting small oil droplets have relatively long lifetime in the water before they were transported to the surface due to buoyancy. For very small droplets, the buoyancy effect might be weak compared to other transport mechanisms in the water column, indicating that very small oil droplets (e.g. 35 μm and smaller) were likely not to surface.

### 3.2 WAF and CEWAF

Excess oil was observed on the surface of the BRT of both WAF and CEWAF tanks. WAF and CEWAF were produced by adding a total of 24 ml (M1) or 25 ml (M2) of Macondo Surrogate oil or oil plus Corexit which is equivalent to the addition of 20.6 gm (M1) to 21.5 gm (M2) of Macondo Surrogate oil and 1.14 g (M1) to 1.19 g (M2) of Corexit 9500 to 130 L of water in the BRT. The highest possible concentration if 100% dispersion occurred would have been 158 mg/l (M1) and 165 mg/l (M2) of Macondo Surrogate oil. However, the WAF EOE concentration for M1 and M2 were 3.6 and 0.26 mg/l, respectively. Only a small amount of the oil added to the BRT remained in the water. The reason for higher energy stirring of M1 was to produce higher concentrations of WAF which resulted in 10 times higher concentrations of WAF than recorded in M2. These WAF EOE concentrations were in the upper range of oil concentrations reported during and a few months after the DWH oil spill where only 5% of over 20,000 sample concentrations were above 0.25 mg/l (Wade et al., 2016).

The CEWAF EOE concentration for M1 and M2 were 36.0 and 41.5 mg/l, respectively. The addition of Corexit to the oil resulted in about 25% of the oil added to the BRT being present in the water. In the CEWAF treatment the highest possible Corexit 9500 (specific gravity 0.949 g/ml; USEPA, 1995) concentration if 100% dispersion occurred would have been 8.8 mg/l (M1) to 9.2 mg/l (M2) of Corexit in the water. Corexit concentrations were not measured so this sets a limit on the maximum possible concentrations. The percent effectiveness of Corexit 9500A on South Louisiana Crude oil (e.g. Macondo Surrogate oil) has been reported as 54.7% (USEPA, 1995) in a swirling flask test. These conditions do not represent the BRT as the low energy mixing allowed for resurfacing of the oil. Therefore, we conclude that the actual Corexit content in our experiment was likely to have been <9 mg/l. We did not determine whether the concentration of CEWAF would have been higher if we would have used a 1:10 ratio of dispersant to oil compared to the 1:20 ratio that we used. However the EPA recommend 1:10 to 1:50 so we used a figure in between the suggested ratios and one we had used in other experiments (Knap et al., 1983).

### 3.3 Mesocosms

**Fig. 5:**
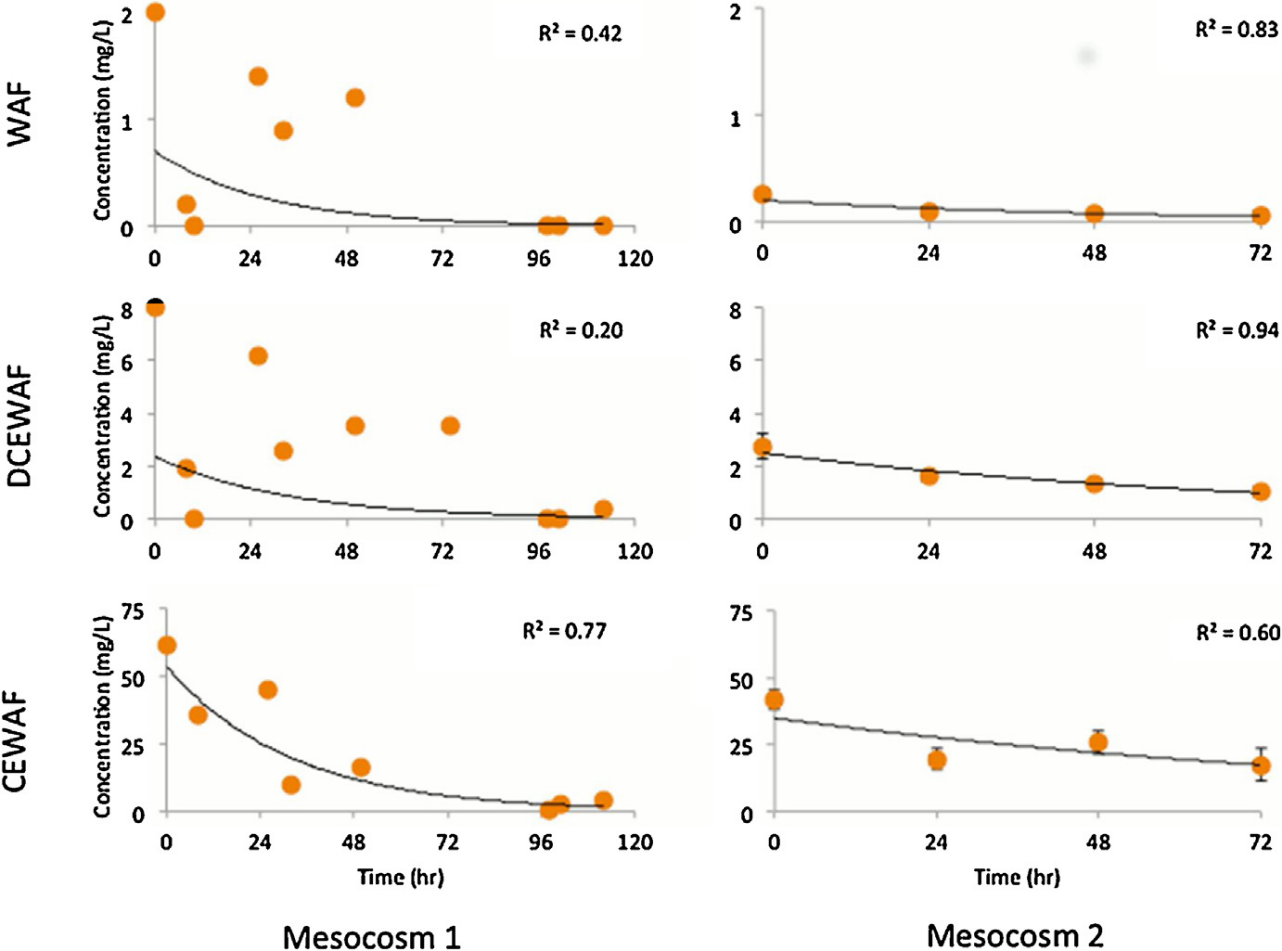
Estimated oil equivalent (EOE) concentrations (mg/l) versus time.

Mesocosm 1: M1 did not have replicate treatments as it was a pilot study and was used primarily to refine the experimental design. However, within 4 h of the start of M1, MOS formed in all treatment tanks, including marine snow in the control (Fig. 2). The EOE concentration of the control, WAF, DCEWAF and CEWAF at the start of the experiments were estimated as 0.00, 3.4, 3.6, and 36 mg/l, respectively (Fig. 5). If all the oil added to the BRT was in the WAF and CEWAF the EOE would indicate that only 2% and 23%, respectively were accommodated in these two treatments. Based on the results of this pilot study the sampling ports were redesigned to be larger in an attempt to decrease variability of EOE due to oil droplets of the samples.

Mesocosm 2: The EOE concentration for the control, WAF, DCEWAF and CEWAF at the start of the experiments were 0.00, 0.26, 2.74, and 41.5 mg/l, respectively (Table 2). Compared to the maximum possible concentration produced if all the oil added to the BRT were in the WAF and CEWAF the EOE indicate only 0.2% and 26%, respectively were accommodated in the mesocosms similar to M1. The total amount of oil accommodated in the WAF was higher in M1 due to the periodic higher energy stirring however this led to more variability at the various sampling plans as the oil had far more droplets and if they were sampled at each time point this resulted in greater inhomogeneity (Fig. 5). EOE values decreased during all treatments, but was more pronounced in the WAF mesocosm tanks during the first 20 h, where EOE values decreased three-fold, from 0.3 to 0.1 mg/l. The EOE average concentration of the control, WAF, DCEWAF and CEWAF mesocosms after 72 h were 0.00, 0.06, 1.03, and 17.3 mg/l, respectively. The percent relative standard deviations ranged from 3.8 to 42% within the range reported for other mesocosom studies (Gearing et al., 1979).

Results for EOE for both M1 and M2 exhibit high variability as the tanks were not stirred due to concerns of breaking the biologically produced strands. This was expected with the presence of droplets, formation of MOS particles of varying sizes, and possible vertical gradients in the mesocosms. Additional variability in microbial biomass, EPS formation and hydrocarbon metabolism likely contributed to the observed variability in the EOE concentrations.

### 3.4 EOE depletion rates

The mean EOE concentrations versus time for the WAF, DCEWAF and CEWAF treatments for M1 and M2 (EOE concentrations) were plotted with an exponential line (Fig. 5). An exponential scale was used as the oil removal process is assumed to be first order. The exponential line fit for the data have determination coefficients (R^2^) ranging from 0.51 to 0.94, reflecting the heterogeneity of the data. All treatments show a decrease in EOE with time. The decrease ranged from −0.013 to −0.027 per hour. These rates are slightly higher than the rate of −0.0066 per hour reported for the MERL mesocosm (Gearing et al., 1979). The reported concentration of the # 2 fuel oil they used for dosing was also slightly lower, at 0.144 mg/l. The MERL study found that while oil was not detected in the water two weeks after oil additions stopped (Gearing et al., 1980), oil was detected in the sediment of the MERL tanks. An estimated 10 to 20% of the #2 fuel oil was deposited in the sediments (Gearing et al., 1980; Wade and Quinn, 1980). Biodegradation was also found to be a significant removal process (Gearing et al., 1979), with different rates for different oil components. Oil in the mesocosms undergoes some of the other processes as in the ocean, i.e. microbial degradation, evaporation, sedimentation, and photo-oxidation (Overton et al., 2016).

### 3.5 Results of PAH analysis

**Fig. 6:**
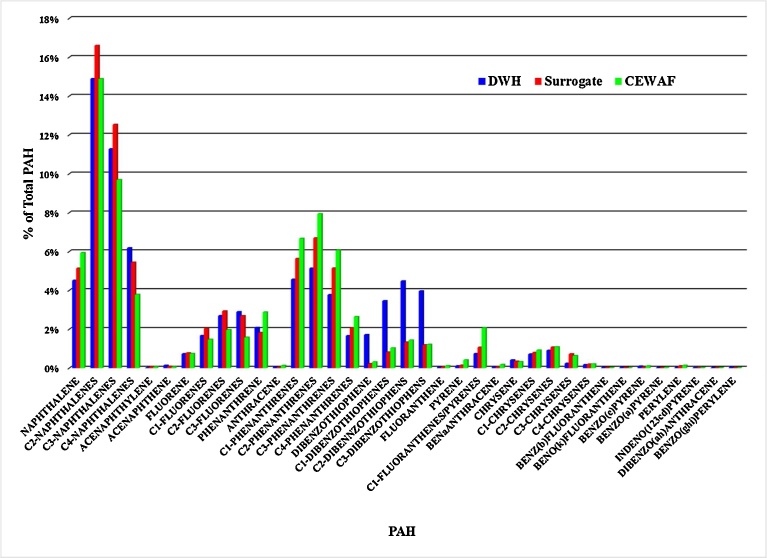
Comparison of GC/MS data of aromatic compounds composition between the original oil from the Deep Water Horizon, the Macondo surrogate oil and the chemically dispersed oil with Corexit 9500A used in this study.

EOE is a rapid and sensitive method to estimate the concentration of oil in water extracts. The main fluorescing components of oil are the PAH. As oil weathers the measurement of fluorescence may overestimate the total amount of oil remaining as aliphatic hydrocarbons are likely to be preferentially metabolized. The PAH distribution for Deepwater Horizon oil (Wade et al., 2016), the Marlin surrogate oil (MSO) and CEWAF at time 0 are shown in Fig. 6. Our analysis of the MSO PAH distribution as a percent of the total PAH shows it is very similar to the DWH oil for most PAH. Both exhibit the typical PAH distribution of an oil (Overton et al., 2016). There are some differences for example higher percentages benzothiophene and the C1-, C2-, and C3- dibenzothiophenes are found in relative to the surrogate oil. The PAH distribution in our CEWAF at time 0 and the MSO are very similar (Fig. 6). The PAH in the WAF and the DCEWAF at time 0 had reduced levels of the napthalene and C1- to C4- naphthalenes (not shown) compared to the CEWAF and MSO. Presumably due to the loss of these more volatile PAH during making of the WAF and DCEWAF production. The PAH distributions produced in the BRT are a valid reflection of what would be expected to be present during an actual oil spill (Wade et al., 2016).

### 3.6 Mesocosm comparative studies

Mesocosms have been used in many oil spill related studies in the past with various scales, from large purposeful spills (Ballou et al., 1987a) such as roller tables and tables and toxicity tests using beakers (Aurand and Coelho, 2005). All mesocosm experiments have limitations, but nonetheless there appears to be a commonality in terms of similar oil concentrations used and ability to illucidate various mechanisms under controlled conditions. The Controlled Ecosystems Pollution Experiment (CEPEX) (Menzel and Case, 1977) was one of the first mesocosm systems consisting of plastic bags floating in cages in Patricia Bay, Nova Scotia. The plastic bags were 2.4 × 13.6 m and contained 66 m^3^ of stratified seawater. One bag was a control, one had 20 g of Exxon Corexit 9527 and the third had 200 g of Prudhoe Bay crude oil and 20 g dispersant, and ^14^C labeled hexadecane. The nominal concentration for the oil if completely dispersed would have been 3 mg/l. As the oil was not evenly distributed in the mesocosms, the measured concentrations over time ranged from 0.05 to 4.2 mg/l in the particulate phase, and 0.2 to 4.5 mg/l in the dissolved phase. The experiment ran for 25 days with daily sampling for many parameters including hydrocarbons (Whitney, 1984). Conclusions stated that oil plus Corexit had a significant effect on phytoplankton species succession, primary productivity, nutrient uptake rates and nitrogen substrate preference, while Corexit alone had no significant effect (Harrison et al., 1986). Centric diatoms were most sensitive to the oil dispersant mixture, while microflagellates were least sensitive.

One of the recognized shortcomings of the CEPEX experiment was that there was not a benthic component, and the bags were static and not flow-through. This was addressed in the MERL (Marine Ecosystems Research Laboratory) tanks developed at the University of Rhode Island in the 1970s. The MERL tanks consisted of twelve fiberglass tanks which were 5.5 meters high and 1.8 m in diameter containing 13 m^3^ of water in a flow-through system. In addition 0.8 m^3^ of silty-clay sediments with associated organisms were collected from Narragansett Bay. In experiments described by Gearing et al. (1979) six tanks were used and the oil was introduced as an oil in water dispersion, which was made by shaking a known amount of No. 2 fuel oil with water for 5 min and creating a mixture of about 63–220 mg/l, or an average of 115 mg/l, and then added to the mesocosms. Three experiments were carried out for different periods of time. The flow rate of the tanks was 330 ml/min to match the residence time of the water in Narragansett Bay (27 days). In experiment 1, a single dose of oil was added and followed for a month. Forty-five samples were taken for hydrocarbon analysis, which averaged 8.5 μg/l with a standard deviation of 6.5 μg/l or 76% relative standard deviation (Gearing et al., 1979). This shows the heterogeneity of oil-in-water mixtures. In experiment 2, oil was added semi-weekly and the experiment ran for 24 weeks, with an average water column concentration of 190 μg/l. The third experiment was for 16.5 weeks and the oil added in semi-weekly additions and averaged 93 μg/l.

Knap et al. (1983) created a flow through system to dose corals in a set of 16 mesocosm tanks. The corals were collected in the field, and 15 corals were placed in each of the 120 dosing mesocosms. Oil and chemically dispersed oil were produced in a baffled flume with 9 chambers and introduced to the tanks over a 24 h period continuously at 3 l/min. This scenario was chosen to represent a 24 h, one time, oiling of a reef. A live Arabian light Crude oil was applied; other treatments were Arabian light crude with Corexit 9527 (1:20) and Arabian Light with BP 1100WD dispersant (1:10). Each of the tanks were sampled, the EOE concentrations measured by fluorescence every 30 minutes. The oil concentrations were adjusted by increasing or decreasing the WAF or CEWAF addition, to meet a 20 mg/l target in continuous dosing. The concentrations with the oil only ranged from 10–30 mg/l, and the Exxon 9527-dispersed oil ranged from 8–40 mg/l. At the end of the 24 h, the corals were treated with fresh seawater for 48 h and then transported to the reef by boat and cemented to the offshore reefs for long-term growth studies (Dodge et al., 1984; Knap et al., 1985). Only the continuous measuring of the EOE concentrations in each tank and the subsequent adjusting of the concentration allowed for an accurate assessment of the dosing.

An experimental system was designed by Nordtug et al., 2011 to produce realistic concentrations of dispersed oil at 2.5 mg/l, 0.8 mg/l, and 0.25 mg/l continuous introduction into 14 (5 L) experimental chambers to primarily investigate the effects of droplet dispersions on toxicity of oil to organisms. This sophisticated system produced reproducible concentrations and droplet sizes used to determine the relative contribution of the dissolved and dispersed oil phases to the overall toxicity of the oil (NRC, 2005).

Perhaps one of the largest “Bay” experiments was carried out on the Panama coast in 1984, where a real test of Net Environmental Benefit Analysis (IPIECA, 1992) occurred, that is, a test of whether one should treat a spill with chemical dispersants (Ballou et al., 1987a; 1987b). Known as the Tropics Experiment, three 10,000 m^2^ sites were selected as a reference and two treated with Prudhoe Bay Crude oil (site O) and Prudhoe Bay Crude oil chemically dispersed with Corexit 9527 (site D). Oil was released over the coral reef, seagrass and mangrove ecosystems over 24 h for site O and 48 h for site D. The oil was monitored by fluorescence continuously, and water column concentrations were targeted at 50 mg/L for the chemically dispersed site, and 1–4 mg/L for the oil only site. The sites were surrounded by an oil boom, and surface oil removed after the dosing periods. The experiment was followed for two years and in addition the site has been periodically re-visited. The last time point of 32 year post application was 2016. Some of the early results are presented in Ballou et al. (1989) and Knap (1987). A term, called “ppm hours” (concentration in mg/l x 24 or 48 h) was used in these experiments. The target concentration was 1200 ppm h for the dispersed site, but actually was 20% higher at 1470 ppm h. Low molecular weight hydrocarbons were higher at the dispersed site (293–684 μg/L) than at the oil only site. The oil site had thick slicks and oil concentrations were between 1–4 mg/L, with exposure at 65–165 ppm h. Low molecular weight hydrocarbons were also collected for the oil only site and were between 33–46 μg/l. Three days after the end of oil dosing, high volume samplers yielded about 10 μg/l at both sites (Ballou et al., 1989).

Various methods for preparing smaller scale WAF have been used, from just shaking oil and water and using large amounts of oil. The Chemical Response to Oil Spills Ecological Effects Research Forum (CROSERF) has defined water-accommodated fraction (WAF) as “a laboratory-prepared medium derived from low energy (no vortex) mixing of oil, which is essentially free of particles and bulk material” (Aurand and Coelho, 2005). To that end, in order to have a consistent preparation of WAF, it is necessary to have a well-established mixture of seawater, mixing energy, and duration of mixing (Singer et al., 2000). The method has been used to typically produce from 1 to 20 L of WAF and CEWAF (Singer et al., 2000). For the present study the CROSERF method would produce stable concentrations of WAF and CEWAF, but would not have produced enough solution quickly enough for fill twelve 110L mesocosms needed for these experiments thus the need for the BRT.

### 3.7 Environmental relevance of the oil concentrations of these experiments

The experiments were designed to mimic realistic oil concentrations found during an actual oil spill as closely as possible. Unfortunately, when an oil spill occurs rarely are there sampling and measurement tools available immediately, especially for an offshore spill. During the DWH there were many measurements (over 20,000) taken near the wellhead and at the surface where the concentrations were high and decreased over time and with distance from the spill site however samples were not taken until at least a week after the initial leakage of oil (Wade et al., 2016). There were very few pre-spill measurements. Yet, estimated background total petroleum hydrocarbon (TPH) concentrations ranged from 1 to 75 μg/l (Wade et al., 2016). The highest concentration, collected near the wellhead was 7,270 mg/l; however only 5% of the samples had concentrations above 0.25 mg/l (Wade et al., 2016). There are few articles on chemically dispersed oil under slicks. Kennicutt et al. (1991) and Payne et al. (1993) studied the Mega Borg spill, where a light Angola Planca Crude was spilled in the Gulf of Mexico off Texas in 1990. The maximum oil concentration under the center of the slick after dispersant use was 22 mg/l. The oil only concentrations ranged between 1.2 and 3.9 mg/l. In the case of the Sea Empress in 1996, which leaked over 72,000 tons of Forties Blend light crude oil for a period of six days, different dispersants were sprayed over the slick and measurements were made by fluorescence with concentrations about 3 mg/l for oil only, and in the chemically dispersed treatment hydrocarbons were measured to be 3 mg/l to a depth of 5 m. Oil concentrations decreased to 0.5–0.6 mg/l after 4 days and by 12 days they were 0.2 mg/l (Lunel et al., 1997). In a purposeful spill treated with Exxon 9527 and North Slope Crude Oil in coastal Panama, concentrations were estimated to be over 60 mg/l (Ballou et al., 1987a, 1987b).

In a review of the significance of dispersants it was reported that during the first hour of dispersant use on an oil spill the concentration of oil went from 40 to 60 mg/l, to less than 1 mg/l within 2 to 5 h (Lessard and Demarco, 2000; Lee et al., 2013; Berjarano et al., 2014). These rapid losses are due to physical oceanic processes (e.g. advection and surface currents). These CEWAF concentrations are clearly an end member for some of the other biological measurements made, and reflect maximum but realistic concentrations within the first hour of chemical dispersant application (Lessard and Demarco, 2000). Concentrations for CEWAF only in our mesocosms are thus in the high end of a reasonable range to mimic actual concentrations of oil and dispersents found during an oil spill. It is important to emphasize that our experiments were not designed for toxicity studies but to elucidate the mechanisms of MOS formation.

## Conclusions

The use of the BRT allows WAF to be produced reliably and efficiently in large volumes. In addition, all of the concentrations of EOE in the WAF and CEWAF treatments are consistent with environmental concentrations (higher range) seen during spills including the DWH. For example, the EOE CEWAF concentrations for field studies range from 10 to 60 mg/l (Lessard and Demarco, 2000; Berjarano et al., 2014) and the CEWAF produced for these experiments was within this range (36 mg/l M1 and 41.5 mg/l M2). The concentration in the field studies decreased rapidly to 1 mg/l or less in 4 h (Lessard and Demarco, 2000; Lee et al., 2013; Berjarano et al., 2014) as expected with the use of dispersants in the ocean with winds and currents. The concentrations in our experiments decrease at a slower rate likely due to the absence of mixing, as mesocosms were purposely not mixed as the objective was to study the biology and chemistry of EPS production. When the plankton mix with associated bacteria were added to the mesocosms, EPS was formed rapidly including in the control tanks. Therefore the production of EPS is a natural process. The high EOE concentration in the CEWAF tank did not inhibit MOS formation and deposition. The produced WAF and CEWAF had variable concentrations (relative percent difference of 3.8 to 41%), but that variability is within the expected range found by others (Lessard and Demarco, 2000; Lee et al., 2013; Berjarano et al., 2014). These mesocosms closely mimic the natural environment, and results from companion ADDOMEx project studies will provide additional insights into the processes including the extent, rate and chemistry of MOS formation as well as oil biodegradation and sedimentation (Quigg et al., 2016). These mesocosms use reasonable EOE concentrations and replicate PAH concentrations in the parent oil (the more toxic components of the oil) while they do not exactly reflect the actual ocean environment they are providing valuable data to advance the understanding of the role of EPS in oil sedimentation and degradation during oil spills.

## Declarations

### Author contribution statement

Terry Wade, Anthony Knap: Conceived and designed the experiments; Performed the experiments; Analyzed and interpreted the data; Wrote the paper.

Dawei Shi, Gerado Gold-Bouchot, Binbin Wang: Performed the experiments.

Maya Morales-McDevitt, Stephen Sweet, Gopal Bera: Performed the experiments; Contributed reagents, materials, analysis tools or data.

Antonietta Quigg: Conceived and designed the experiments; Wrote the paper.

### Funding statement

This work was supported by a grant from The Gulf of Mexico Research Initiative to support the consortium research ADDOMEx (Aggregation and Degradation of Dispersants and Oil by Microbial Exopolymers). Anthony Knap and Gopal Bera were supported by DE-TOX (Deep Sea- Risk Assessment of oil and oil dispersants).

### Competing interest statement

The authors declare no conflict of interest.

### Additional information

Data associated with this study has been deposited at Gulf of Mexico Research Initiative Information and Data Cooperative (GRIIDC), http://data.gulfresearchinitative.org, under the accession number http://dx.doi.org/10.7266/N7TB14ZW and http://dx.doi.org/10.7266/N7PK0D64
